# Taurine Antagonizes Macrophages M1 Polarization by Mitophagy-Glycolysis Switch Blockage *via* Dragging SAM-PP2Ac Transmethylation

**DOI:** 10.3389/fimmu.2021.648913

**Published:** 2021-04-12

**Authors:** Ling Meng, Cailing Lu, Bin Wu, Chunhua Lan, Laiming Mo, Chengying Chen, Xinhang Wang, Ning Zhang, Li Lan, Qihui Wang, Xia Zeng, Xiyi Li, Shen Tang

**Affiliations:** ^1^ School of Basic Medical Sciences, Guangxi Medical University, Nanning, China; ^2^ Guangxi Colleges and Universities Key Laboratory of Preclinical Medicine, Guangxi Medical University, Nanning, China; ^3^ School of Public Health, Guangxi Medical University, Nanning, China

**Keywords:** M1 macrophage, low-grade inflammation, taurine, PP2Ac methylation, SAM, mitophagy

## Abstract

The excessive M1 polarization of macrophages drives the occurrence and development of inflammatory diseases. The reprogramming of macrophages from M1 to M2 can be achieved by targeting metabolic events. Taurine promotes for the balance of energy metabolism and the repair of inflammatory injury, preventing chronic diseases and complications. However, little is known about the mechanisms underlying the action of taurine modulating the macrophage polarization phenotype. In this study, we constructed a low-dose LPS/IFN-γ-induced M1 polarization model to simulate a low-grade pro-inflammatory process. Our results indicate that the taurine transporter TauT/SlC6A6 is upregulated at the transcriptional level during M1 macrophage polarization. The nutrient uptake signal on the membrane supports the high abundance of taurine in macrophages after taurine supplementation, which weakens the status of methionine metabolism, resulting in insufficient S-adenosylmethionine (SAM). The low availability of SAM is directly sensed by LCMT-1 and PME-1, hindering PP2Ac methylation. PP2Ac methylation was found to be necessary for M1 polarization, including the positive regulation of VDAC1 and PINK1. Furthermore, its activation was found to promote the elimination of mitochondria by macrophages *via* the mitophagy pathway for metabolic adaptation. Mechanistically, taurine inhibits SAM-dependent PP2Ac methylation to block PINK1-mediated mitophagy flux, thereby maintaining a high mitochondrial density, which ultimately hinders the conversion of energy metabolism to glycolysis required for M1. Our findings reveal a novel mechanism of taurine-coupled M1 macrophage energy metabolism, providing novel insights into the occurrence and prevention of low-grade inflammation, and propose that the sensing of taurine and SAM availability may allow communication to inflammatory response in macrophages.

## Introduction

Inflammation is an innate host defense mechanism used to fight infections and various types of tissue damage. The inflammatory process is comprised of different stages, from the acute phase to adaptation and resolution. Moreover, inflammation can develop into a chronic disease, acting as a catalyst for the development of many metabolic and age-related degenerative diseases (1). Macrophages are immune cells that play a role in phagocytosis and the elimination of pathogens (2). Macrophages have a high degree of heterogeneity and plasticity. They are polarized into different phenotypes during the infection process: the classic activated M1 type and the alternatively activated M2 type, which coordinate pro-inflammatory and anti-inflammatory repairs during infection to maintain homeostasis (3, 4). The functions of macrophages in different polarization states are closely related to their metabolism. Pro-inflammatory M1 macrophages activated by IFN-γ and Toll-like receptors (TLRs) rely on enhanced aerobic glycolysis initiated by rapid glucose uptake (5). This rapid and direct energy supply is conducive for macrophages to produce inflammatory factors, which have an anti-infection effect (6). However, uncontrolled M1 activation can lead to the development of chronic low-grade inflammation-related diseases, including atherosclerosis, obesity, type 2 diabetes, and amyotrophic lateral sclerosis (ALS) (7–9). Conversely, rewiring metabolism to mitochondrial oxidative phosphorylation reverses pro-inflammatory activation. Therefore, the regulation of energy metabolism represents a viable means for the treatment of inflammatory diseases.

The regulation of macrophages is a complex process, which involves a series of molecular mechanisms such as protein-protein interaction and post-translational modification (10, 11). SAM derived from dietary methionine is a universal direct methyl donor required by various methyltransferases, likely connecting the status of nutrition and chromatin or protein (12). A recent study found that various nutritional supplements such as methionine and serine may be directly related to the macrophage-mediated inflammation driven by LPS (13), which cooperatively regulate the availability of SAM to support histone H3 methylation to produce IL-1 β. Protein phosphatase 2A (PP2A) is one of the few phosphatases that exhibits dephosphorylation, which can regulates broad range of cellular signaling by dephosphorylating specific substrates (14). The methylation at leucine-309 residue of catalytic C-subunit (PP2Ac) is the main modification modulating PP2A activity, which regulate reversible methylation and demethylation modification by leucine carboxy methyltransferase LCMT-1 and methylesterase PME-1 (15). Interestingly, in experimental DSS enteritis and endotoxin models, PP2Ac methylation or up-regulation of PP2Ac levels promoted macrophage-related inflammation (16, 17). The activation of PP2A involves a variety of cell physiological activities, including the regulation of cellular metabolism, apoptosis, autophagy, cell proliferation (18). Recent studies have pointed out that the activation of PP2A can promote autophagy through the death-associated protein kinase 1 (DAPK1)-beclin1 axis and the transcriptional activation of TFEB (19, 20). In parallel, evidence have shown that autophagy activation plays a role in promoting the functional differentiation of different cells *via* mitochondrial-related metabolic transformation (21–23), indicating that autophagy/mitophagy may be involved in the regulation of macrophages M1 polarization. The role of autophagy in inflammatory diseases can be either deleterious or protective, depending on the stimuli (24–27). Therefore, further data is still needed to clarify how PP2Ac methylation, autophagy, and mitochondrial metabolic reprogramming work synergistically in the M1 polarization of conditioned macrophages.

Taurine, as one of the end products of the downstream metabolic transsulfuration pathway of SAM (28), participates in maintaining the homeostasis of cell energy metabolism and anti-inflammatory processes (29–31). Evidence shows that taurine deficiency may result in an imbalance of energy metabolism in the heart, skeletal muscle, liver, and adipose tissue (32). Instead, supplementation of taurine can improve mitochondrial metabolic dysfunction and reduce inflammation in chronic diseases diabetes and COPD (25, 33). Moreover, in high-fat diet-induced obesity and Parkinson’s disease mouse models, taurine has been shown to balance the polarized phenotype of macrophages and reduce inflammatory injury (34, 35). However, the critical mechanisms of taurine in fighting M1 macrophage-related inflammation have yet to be delineated. Here, we investigated the inflammatory response of macrophages challenged by low-dose LPS/IFN-γ to evaluate the regulatory mechanism of taurine against low-inflammatory M1 polarization. We show that this anti-inflammatory pathway of taurine is achieved through metabolic reprogramming mediated by SAM/PP2Ac methylation/mitophagy axis. Notably, this study highlights the potential of taurine nutritional intervention in the prevention of low-grade inflammatory diseases.

## Materials And Methods

### Chemicals

Phorbol 12-myristate 13-acetate (PMA, P8139, purity > 99%), taurine powder (T0625, purity > 99%), ABL127 (SML0294, purity > 98%), Lipopolysaccharides (LPS, L2630), Methionine-free medium (RPMI-1640 medium, R7513), and methionine powder (L-Methionine, M9625, purity > 98%) were purchased from Sigma-Aldrich Inc. (St. Louis, MO, USA). Cell culture medium (RPMI-1640 medium, 3180022) was purchased from Gibco-Life Technologies (Grand Island, USA). FastStart Universal SYBR Green Master Mix (ROX) (4913914001) was obtained from Roche (Basel, Switzerland); Hexokinase (HK) Assay Kit (BC0680), pyruvate kinase (PK) Assay Kit (BC0545), Lactate dehydrogenase (LDH) Assay Kit (BC0685), Pyruvic acid dehydrogenase (PDH) Assay Kit (BC0385) and Neutral Red solution, 0.33% (G1310) were purchased from Solarbio (Shanghai, China); ATP assay kit (S0026), Mito-Tracker Green (C1048), Mitochondrial membrane potential assay kit with JC-1 (C2006), Lyso-Tracker Red (C1046) and α-Tubulin were obtained from Beyotime (Jiangsu, China). Beclin-1, Cryopyrin (NLRP3), PINK1, Parkin, demethylated PP2Ac, methylated PP2Ac, LCMT-1, PME-1, total PP2Ac antibodies were obtained from Santa Cruz Biotechnology (Santa Cruz, CA). Anti-LC3B antibodies were purchased from Abcam Co. (Cambridge, UK). SQSTM1 (p62) and Bafilomycin A1 (BAF) (54645) were purchased from Cell Signaling Technology Co. (Danvers, MA).

### Cell Culture and Treatment

THP-1 cells, human peripheral blood mononuclear cell line derived from acute monocytic leukemia, were obtained from Shanghai Institutes for Biological Sciences (Shanghai, China) and were grown in RPMI-1640 medium with 10% fetal bovine serum and were incubated in an incubator containing 5% CO_2_ at 37°C. Cells were grown up to 80% confluence and were plated into either a 6-well plate or a 96-well plate and were then cultured with PMA (100 nM) for 24 h before exposed to LPS (10 ng/mL) and IFN-γ (20 ng/ml) for 48 h with or without incubation with taurine (40mM/80mM) or ABL127 (0.5 nM)/methionine (10 mg/L). The morphology of THP-1 cells from each treatment group was observed by an inverted microscope (DMi8; Leica, Germany). After the treatment according to the above, the cells were collected for the following analyses.

### Macrophage Phagocytosis Analysis

Neutral red (NR) staining method was used to detect the phagocytic function of macrophages. THP-1 cells were seeded in 96-well plates for 2×10^5^ cells per well. After the treatment according to the above, the culture supernatant was discarded, 200 µL of 0.1% NR reagent was added into each well and incubate at 37°C for 2 h. The cells were washed three times with PBS after pouring the supernatant, then incubated overnight with 200 µL of lysis buffer (the volume ratio of glacial acetic acid to absolute ethanol is 1:1) at 4°C in the dark. Eventually, the absorbance value of each group at 540 nm was determined by plate reader (Protein Simple, USA).

### Measurement of Taurine Abundance

After the treatment according to the above, collect the cells with 0.1% hydrochloric acid, and operate on ice during the whole process, and then was lysed by ultrasonic degradation (AMPL 30%, repeated 10 times each 3-5 s). Each extract was centrifuged at 10,000 g at 4°C for 10 minutes, and the supernatant was transferred to a new tube, an appropriate amount of sulfosalicylic acid was added to the tube, shaken and mixed, and centrifuged as described above. The supernatant was passed through a 0.22 μm organic phase filter and then bottled. The taurine was separated by chromatography on an amino acid analyzer with lithium buffer (SYKAM S-433D, SYKAM, Germany), and the response value was detected according to the ninhydrin method. Two injections were made to each sample, and the standard product file measured at that time was used as the calibration file for sample integration.

### CCK-8 Assay for Screening of Taurine Concentration

The CCK-8 (Dojindo Laboratories, China) was used to determine the optimal concentration of taurine. THP-1 cells were seeded in 96-well plates for 1*10^4^ cells per well. After the treatment according to the above, and were then cultured with taurine (10/20/40/80/120/160 mM) at the same time. 10 μL of CCK-8 (20 μg/mL) was added to each well with 100 μL new culture medium after discarding the supernatant, and incubated at 37°C for 1 hour. Finally, the optical density of the cells was measured with a plate reader (Protein Simple, USA) at a wavelength of 450 nm.

### The alamarBlue Assay

The alamarBlue as a redox indicator can be used to evaluate metabolic function, which is reduced by FMNH2, FADHs, NADH, NADPH and the cytochromes in the electron transport chain (36). Briefly, THP-1 cells were seeded in 96-well plates for 1*10^4^ cells per well. After the treatment according to the above, 100 μL of 20 μg/mL alamarBlue Reagent (Thermo Fisher, USA) was added to each well and then incubated at 37°C for 2 h. Finally, the optical density of cells was determined at wavelengths of 560 nm and 600 nm with a plate reader (Protein Simple, USA).

### Real-Time PCR

THP-1 cells were seeded in 6-well plates for 8*10^5^ cells per well. After the treatment according to the above, total RNA was extracted with TRIzol and reverse transcribed with an 18T oligo primer. Real-time PCR amplification was performed using FastStart Universal SYBR Green Master Mix (ROX) (Roche, Switzerland) and Thermo Stepone plus Real-Time System according to the manufacturer’s protocols. The PCR conditions were 95°C for 10 min, 95°C for 10 s, and 60°C for 30 s for 40 cycles. Relative mRNA gene levels were normalized to the β-actin mRNA level and relative expressions were determined by the 2^-ΔΔCT^ method. Primers were listed in **Table 1**.

**Table 1:** Sequences.

Gene	5′-3′ Forward	5′-3′ Reverse
**ACTB** **COX2** **TNF** **IL6** **CXCL10** **IL10** **CD206** **SLC6A6** **CDO1** **CSAD** **MAT2A**	TCCTCTCCCAAGTCCACACAGG GCCATGGGGTGGACTTAAATCATA GAGGCCAAGCCCTGGTATG ACTCACCTCTTCAGAACGAATTG TCTCCCATCACTTCCCTACA CCCTTTGCTATGGTGTCCTT AAAC ACAGACTGACCCTTCCC CGTACCCCTGACCTACAACAAA ACCAACTCCCACTGCTTTCTGAA GGAAGCAGGAGAGTCCAGATTACC CCGACACCAACATGAACGGA	GGGCACGAAGGCTCATCATTC CAGGGACTTGAGGAGGGTAGATCA CGGGCCGATTGATCTCAGC CCATCTTTGGAAGGTTCAGGTTG CAGGGTCAGAACATCCACTA TGGTTTCTCTTCCCAAGACC GTTAGTGTACCGCACCCTCC CAGAGGCGGATGACGATGAC ACCAACTCCCACTGCTTTCTGAA GCCACAACCACACGGAAGAAG AGCAACAGTTTCACAAGCTACT

### Western Blot

After following the above treatment, total protein was extracted with cold RIPA medium lysis buffer containing protease inhibitor (P0013C, Beyotime, China) and 1 mM PMSF, the extract was kept on ice for 20 min and then centrifuged at 4°C (12000 rpm/min, 15 min). The supernatant was collected and protein content was detected by the bicinchoninic acid (BCA) protein assay kit (TaKaRa, Japan) according to the reagent Instructions. Equal amounts of protein were separated by SDS-polyacrylamide gel and then transferred to PVDF membrane. Membranes were incubated overnight at 4°C with the primary antibodies and then by horseradish peroxidase-linked goat anti-rabbit IgG (1:2000) or anti-mouse IgG (1:2000) for 2 h at 25°C. The iBright imaging system (FL1000, Thermo Fisher, USA) were used for the acquisition and analysis of western blot images.

### Measurement of HK, PK, LDH, and PDH Activity

THP-1 cells were seeded in 6-well plates at a density of 1 × 10^6^ cells per well. After treatment, the cell extracts were scraped on ice using a cell scraper with glycolysis-related enzymes and pyruvic acid dehydrogenase (PDH) extraction reagent, and then lysed by ultrasonic degradation (AMPL 20%), repeated 30 times every 3 s. Each extract was centrifuged at 8000 × *g* for 10 min at 4°C. The corresponding reagents were added according to the manufacturer’s instructions and incubated at 37°C for the specified time. The absorbance values (A1/A2 and A3/A4) at 340 nm (HK, PK)/450 nm (LDH)/605 nm (PDH) were detected using a plate reader (Protein Simple, USA). U/10^4^ cell (HK, PK) = [ΔA × V_total reaction_ ÷ (ϵ × d) × 10^9^] ÷ (500 × V_sample_ ÷ V_extract_) ÷ T, U/10^4^ cell (LDH) = y × V_sample_ ÷ (500 ÷ V_extract_ × V_sample_) ÷ T × 10^3^, and U/10^4^ cell (PDH) = [(ΔA_sample_ – ΔA_black_) × V_total react ion_ ÷ (ϵ × d) × 10^9^] ÷ (V_sample_ ÷ V_extract_ × 500) ÷ T. For HK, the corresponding amount of reagent was added according to the kit instructions. Then, 10 μL of sample supernatant was added, and the absorbance was recorded A1 at 340 nm for 20s. The absorbance A2 was recorded after 5 minutes of incubation, and used to calculate ΔA = A2–A1. The total enzyme activity unit was calculated according to the following formula: HK (U/10^4^ cell) = 2.143 × ΔA. For PK, the corresponding reagent was added according to the kit instructions. Then, 10 µL of sample supernatant was added, and the absorbance A1 was recorded at 340 nm for 20 s. The absorbance A2 was recorded after 2 min of continuous incubation and used to calculate ΔA = A1–A2. The enzyme activity was calculated according to the following formula: PK (U/10^4^ cell) = 5.36 × ΔA. For LDH, the sample, control, and standard tubes were set up (100 μL of standard solution diluted to 1.0, 0.5, 0.25, 0.125, and 0 μmol/mL in multiple ratio) with a volume of 10 μL. The corresponding reagent was added according to the instructions, and mixed well. Then, 200 μL of the mixture was transferred to a 96-well plate, and the absorbance was measured at 450 nm. ΔA was calculated as A_sample_ – A_control_, and used to draw the standard curve, with ΔA as *y*. The enzyme activity was calculated according to the following formula: LDH (U/10^4^ cell) = 0.133 × *y*. For PDH, the sample and blank tubes were set up. Briefly, 180 µL of working solution was incubated at 37°C for 10 min in each tube (prepared according to the instructions). Then, 10 μL of sample supernatant (blank tube added with 10 μL water) was added, and the initial absorbance value at A1_blank_/A3_sample_ was measured at 605 nm. The absorbance value A2_blank_/A4_sample_ was measured after 1 min, and used to calculate ΔA_blank_ = A1–A2 and ΔA_sample_ = A3–A4. The enzyme activity was calculated according to the following formula: PDH (U/10^4^ cell) = 3.655 × (ΔA_sample_–ΔA_control_).

### ATP Measurement

An ATP detection kit was purchased from Beyotime (Jiangsu, China). THP-1 cells were seeded in 6-well plates at 1 × 10^6^ cells per well. After the above treatment, 200 µL of lysate was added and placed on ice for lysis. Then, the cells were collected into a 1.5-mL EP tube. After centrifugation at 12000 × *g* for 5 min at 4°C, the supernatant was collected, and the detection wells and standard wells were set up (standard solution concentrations of 0.01, 0.03, 0.1, 0.3, 1, 3, and 10 μM). Next, 100 µL of ATP detection solution was added to the 96-well plate and allowed to stand at room temperature for 3-5 minutes. Then, 20 µL of sample supernatant was added to the detection wells and standard wells, shaken, mixed, and immediately evaluated using a plate reader (Protein Simple, USA) to determine the RLU value. Lastly, a standard curve was drawn and used to calculate the ATP concentration.

### Fluorescence Staining and Co-Localization Analysis of Mitochondria and Lysosomes

To detect fluorescence signal intensity of mitochondria and lysosomes, cells were seeded in the 48-well or 96-well plates and treated as above, and PBS washing twice before were cultured with 10 nM lyso-Tracker Red (C1046, Beyotime) and Mito-Tracker Green (C1048, Beyotime) at 150 nM for 30 min at 37°C. The results were analyzed by using automatic fluorescence imaging system (Evos FL, Thermo Fisher, USA) and the fluorescence intensity was read by a multifunction microplate reader (ProteinSimple, USA) at excitation wavelength of 590 nm (mitochondria: 490 nm) and emission wavelength of 635 nm (mitochondria: 535 nm). The co-localization of mitochondria and lysosomes was detected by using scanning confocal microscope (Zeiss LSM 510, Carl Zeiss, Germany) and analyzed by imaging processing software (ZEN, Zeiss and image J). The M1 group was set as the reference signal, and the contribution of the green (mitochondria)/red (lysosome) to the colocalization of the both signals was quantified as the overlap coefficient (Kx-green)/(Kx-red).

### Measurement of Intracellular ROS Levels

The treated cells were washed with PBS twice, and then tested by a 2,7-dichlorofluorescein diacetate (DCFH-DA) assay (Beyotime, China), set up a positive control group (Rosoup reagent) before adding DCFH-DA and incubate at 37°C for 20 minutes. After discarding the supernatant and washing twice with PBS, the cells were incubated with DCFH-DA (10 μmol/L) at 37°C for 30 minutes. The fluorescence intensity was read by a plate reader (Protein Simple, USA) (excitation wavelength of 488 nm and emission wavelength of 525 nm).

### Measurement of Mitochondrial Membrane Potential Levels

The mitochondrial membrane potential levels were tested by JC-1 assay kit (Beyotime, China). THP-1 cells were seeded in 48-well plates for 3×10^5^ cells and washed twice with PBS. The positive control group (1 μM CCCP reagent) was established before JC-1 was added and incubated at 37°C for 20 minutes. Each group was washed twice with JC-1 staining buffer (1×), incubated with JC-1 (1×) at 37°C for 30 minutes, and then washed twice with ice JC-1 buffer. The mitochondrial membrane potential fluorescence images were collected by Evos FL automatic fluorescence imaging system.

### UPLC-MS/MS Analysis

SAM and SAH were determined by ultrahigh-performance liquid chromatography tandem mass spectrometry (UPLC-MS). The counted cells were collected in 1.5-mL Eppendorf tubes containing 200 μL of the initial mobile phase (5.0 mmol/L ammonium acetate + 0.4% acetic acid + 2% methanol) and repeatedly frozen and thawed at -80°C three times to break the cells. Then, all samples were clarified by centrifugation at 10000 × g for 10 minutes at 4°C. The supernatant was taken and brought to volume at 1 mL, passed through an organic phase filter (0.22 μm, 13 mm), and tested within 24 hours after bottling. The levels of SAM and SAH were assayed using a UPLC system (CORTES, Waters Corp, USA) interfaced with a mass spectrometer (QTRAP 4500, AB Sciex, USA). Briefly, the samples were separated on a CORTES UPLC-C18+ column and subjected to multiple reaction monitoring (MRM) scanning in electrospray ion source positive ion (ESI+) mode. The column temperature was maintained at 40°C, the flow rate was 0.35 mL/min, and the gradient elution process was as follows: buffer A: 0.4% (v/v) acetic acid aqueous solution containing 10 mmol/L ammonium acetate; buffer B: methanol. T = 0 minutes, 0% B; T = 1 minute, 2% B; T = 3 minutes, 75% B; T = 4 minutes, 2% B; and T = 5 minutes, 2% B. The ESI source voltages were 5.5 kV with a capillary temperature of 400°C; CUR: 206 kPa; collision gas (CAD): medium; GS1: 384 kPa; and GS2: 384 kPa. The injection volume was 2 µL, and triplicate injections were performed for each sample. The area under each peak was quantified using software, and the accuracy was re-examined.

### Statistical Analysis

All data are presented as means ± SD. The statistical analyses were performed using SPSS 22.0 statistical software (SPSS Inc., Chicago, IL, USA). Statistical graphs were generated using GraphPad Prism 8 statistical software (GraphPad Software Inc., San Diego, CA, USA). One-way ANOVA was used to determine the differences among groups, followed by LSD’s *post hoc* test to determine the differences between two groups. The difference was considered significant when p < 0.05.

## Results

### Activation of Taurine Synthesis and Transport in Macrophage M1 Polarization

**Figure 1:**
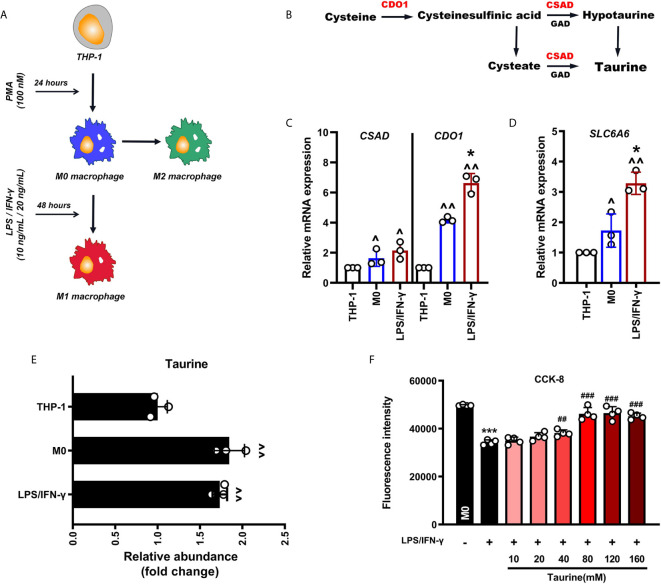
Taurine synthesis and transport are up-regulated at the transcriptional level in LPS/IFN-γ-challenged macrophages. **(A)** Schematic representation of macrophage polarization. **(B)** Schematic representation of the synthesis of endogenous taurine. **(C, D)** *CDO1*, *CSAD*, and *SLC6A6* mRNA levels in M1 polarization of macrophages, n=3. **(E)** The abundance of taurine in M1 polarization of macrophages, n=3. **(F)** CCK-8 analysis in taurine dosage selection, n=4. ^p < 0.05 and ^^p < 0.01 vs. THP-1; *p < 0.05 and ***p < 0.001 vs. M0; ##p < 0.05 and ###p < 0.001 vs. LPS/IFN-γ.

We constructed a pro-inflammatory polarization model of M1 macrophages induced by LPS and IFN-γ *in vitro*. THP-1 cells were treated with PMA (100 nmol/L) for 24 h to activate M0 macrophages, and then challenged with LPS (10 ng/mL) and IFN-γ (20 ng/mL) for 48 h to induce the polarization of M0 to M1 pro-inflammatory macrophages (**Figure 1A**). To determine whether taurine is involved in the M1 polarization of macrophages, we first detected the mRNA expression of the taurine rate-limiting enzymes cysteine dioxygenase type I (*CDO1*), cysteine sulfonic acid decarboxylase (*CSAD*), and the taurine transporter TauT (*SLC6A6*) (**Figure 1B**). We found that the expression of *CDO1*, *CSAD*, and *SLC6A6* continued to increase during activation and polarization (**Figures 1C, D** **)**. An amino acid analyzer was used to measure the intracellular taurine abundance. The results showed that the response value of taurine abundance increased by 1.6-times during the activation of THP-1 to M0. However, there was no upward trend compared with M0 after further polarization to M1 (**Figure 1E**). These data suggest that intracellular taurine synthesis and transport are active during M1 polarization of macrophages, but they do not affect the abundance of intracellular taurine.

### Taurine Inhibits the Polarization of Macrophages to M1 and Increases the Expression of M2-Like Markers

Since taurine in tissues and cells is highly dependent on external uptake, we speculated that the constant intracellular taurine level during M1 polarization may result from the lack of taurine in the incubation environment. To determine whether taurine supplementation affects M1 polarization, we observed the morphological changes in M1 polarization and the mRNA expression of COX-2, TNF-α, IL-6, and CXCL-10 after treatment with taurine. The dose of taurine was chosen based on the CCK-8 assay (40 mM/80m M) (**Figure 1F**). Since taurine is not cytotoxic over a wide range, we decided to evaluate the protective effect of different concentrations of taurine on the viability of LPS/IFN-treated macrophages. The results showed that taurine significantly promoted the viability of LPS/IFN-γ-treated macrophages in a dose-dependent manner. The difference started to be significant at a concentration of 40 mM, and was highest at 80 mM, showing a tendency to be stable at higher concentrations. Therefore, in this model, 40 mM and 80 mM were selected as the doses of taurine.

**Figure 2:**
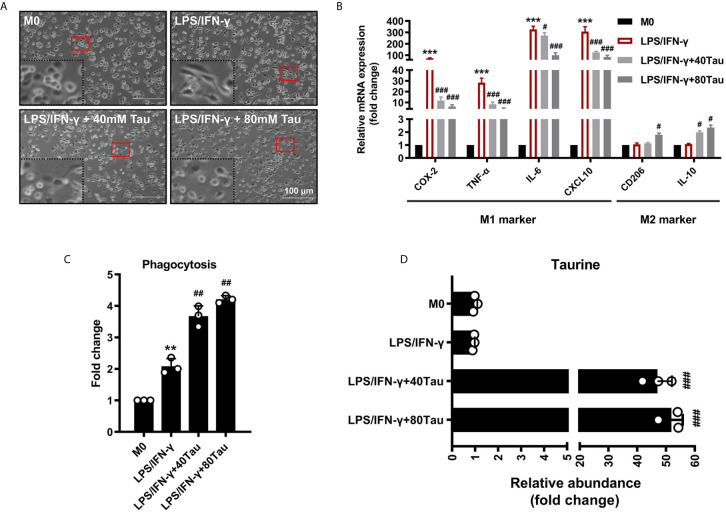
Taurine supplementation attenuates macrophages M1 polarization. **(A)** The effect of taurine on morphology of M1 macrophages polarization. **(B)** M1 and M2 marker mRNA levels in M1 polarization with taurine treatment. **(C)** Effect of taurine on phagocytosis of macrophage. **(D)** Taurine abundance in M1 macrophage polarization after exogenous taurine intervention. n=3. **p < 0.01, ***p < 0.001 vs. M0; #p < 0.05, ##p < 0.01 and ###p < 0.001 vs. LPS/IFN-γ.

The polarization of macrophages to the M1 pro-inflammatory phenotype can cause morphological changes in macrophages and the production of a large number of pro-inflammatory cytokines (37). As shown in the results, the mRNA levels of COX-2, TNF-α, IL-6, and CXCL-10 in M1 macrophages increased. Compared with the M1 group, we observed that, in the taurine treatment group, the number of spindle-shaped M1 macrophages decreased, the pseudopod-like ridges on the surface disappeared and showed round-like changes, and the above M1 gene expression was markedly decreased (**Figures 2A, B** **)**. Phagocytosis is the basic mechanism by which macrophages exert immune function, and M1 and M2 can both show active phagocytosis in different inflammatory reactions. Interestingly, we found that while taurine intervention reduced the expression of M1 inflammatory factor genes, it also increased the expression of M2 related genes CD206 and IL-10, and further increased phagocytosis (**Figures 2B, C** **)**. The data of amino acid analysis also showed that exogenous taurine supplementation markedly increased the taurine abundance in macrophages in a dose-response relationship, by 53.4- and 61.0-times, respectively (**Figure 2D**). Our data suggest that exogenous supplementation satisfies the taurine demand of LPS/IFN-γ-challenged macrophages to avoid excessive pro-inflammatory polarization and orchestrate macrophage activation from pro-inflammatory M1 toward anti-inflammatory M2.

### Taurine Reprograms the Energy Metabolism of Macrophages

#### Enriched Taurine Eliminates The Characteristics of Glucose Metabolism Required by M1 Macrophages

**Figure 3:**
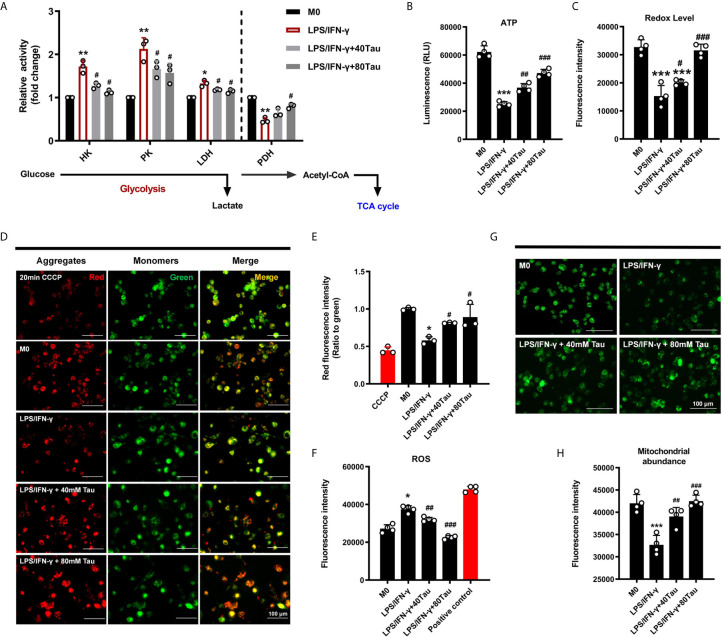
Taurine reprograms the energy metabolism of macrophages. **(A)** Activities of glycolysis enzyme and PDH, n=3. **(B)** The levels of ATP, n=4. **(C)** alamarBlue assay, n=4. **(D–F)** Taurine up-regulates mitochondrial membrane potential and reduces the level of ROS in LPS/IFN-γ-challenged macrophages, n=4. **(G, H)** Fluorogram and quantitative analysis of mitochondrial abundance, n=4. *p < 0.05, **p < 0.01 and ***p < 0.001 vs. M0; #p < 0.05, ##p < 0.01 and ###p < 0.001 vs. LPS/IFN-γ.

Similar to the Warburg effect in tumor cells, mitochondrial oxidative phosphorylation (OXPHOS) levels are reduced and aerobic glycolysis is enhanced, which is a characteristic of glucose metabolism in M1 macrophages, requiring rapid and small amounts of ATP supply to support their function (6). However, changes in the metabolic program can cause a shift in the polarized phenotype (M1/M2) of macrophages (38, 39). To evaluate the effect of taurine on glucose metabolism in M1 polarization, we first detected the activities of key enzymes involved in glycolysis and mitochondrial energy metabolism. The data showed that when M0 was polarized to M1 macrophages, the activities of glycolysis enzymes HK, PK, and LDH all showed an increasing trend, and the activity of PDH, the rate-limiting enzyme of the tricarboxylate (TCA) cycle, decreased markedly. However, compared with M1, taurine treatment groups showed a lower glycolytic enzyme activity and higher PDH activity (**Figure 3A**). In addition, M1 macrophages showed a sharp decrease in intracellular ATP levels, whereas taurine supplementation increased ATP levels in a dose-dependent manner (**Figure 3B**). The switch of the dominant glucose metabolism pathway leads to fluctuations in ATP levels. Similarly, the results of the resazurin test showed that taurine supplementation prevented the low redox state in M1 macrophages (**Figure 3C**). These data suggest that enriched taurine eliminated the metabolic characteristics required by M1 macrophages and blocked the energy metabolism from inclining to glycolysis under the LPS/IFN-γ challenge.

#### Taurine Prevents the Loss of Mitochondrial Membrane Potential and Abundance Induced by LPS/IFN-γ

In cell differentiation, mitochondria, which are the central organelle responsible for stress adaptation and energy homeostasis, undergo biological profound changes to maintain the phenotype of immune cells (40). In the present study, the ratio of red to green fluorescence of JC-1 dye was used to detect changes in the mitochondrial membrane potential (MMP, ΔΨm). JC-1 aggregates and emits red fluorescence in normal mitochondria, but exists as a green fluorescent monomer during depolarization. The ratio of red to green fluorescence decreased in cells treated with LPS/IFN-γ for 48 h, indicating that there was less aggregation in mitochondria and reduced MMP, while taurine supplementation increased it (**Figures 3D, E** **)**. At the same time, taurine was found to reduce the cellular ROS levels (**Figure 3F**).

Cells can respond to changes in metabolic needs in a timely manner by regulating the mitochondrial number (41). We fluorescently labeled the mitochondria to analyze the changes in their content. As shown in the results, the green fluorescence intensity decreased markedly after LPS/IFN-γ treatment, suggesting that the mitochondrial content in M1 macrophages was greatly reduced. Enriched taurine blocked the degradation of mitochondria under pro-inflammatory conditions to a large extent (**Figures 3G, H** **)**. These data suggest that the addition of taurine prevents the shift of metabolic mode to glycolysis to inhibit M1 polarization, which may be achieved by maintaining the steady state of mitochondrial quality and quantity.

### Mitophagy Blocking Is Responsible for the Metabolic Reprogramming Effect of Taurine on Macrophages

**Figure 4:**
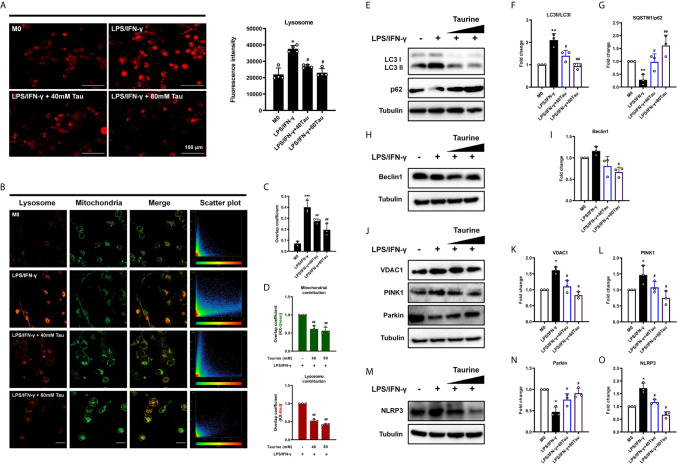
Taurine blocks mitophagy in LPS/IFN-γ-challenged macrophages. **(A)** Fluorogram of lysosomes in M1 macrophage and its quantification analysis, n=4. **(B)** Colocalization analysis of endocytic probes. Mitochondria (green), lysosome (red) and the merged images, and the scatterplot of red and green pixel intensities. Scale bar, 20 μm. **(C)** Pearson’s correlation coefficients (PCCs) of merged images, n=3. **(D)** Quantification of the overlap coefficient (Left: Kx-green/Right: Kx-red) in the merged images, n=3. **(E–O)** Representative immunoblots and quantitation of LC3 II/I, p62, Beclin-1, VDAC1, PINK1, Parkin, and NLRP3, n=3. *p < 0.05, **p < 0.01 and ***p < 0.001 vs. M0; #p < 0.05 and ##p < 0.01 vs. LPS/IFN-γ.

Mitophagy maintains cell homeostasis and functions in cell differentiation and development, immune responses, and cell programming. Mitophagy is considered to be the key to the metabolic shift in cell fate transition during differentiation and dedifferentiation (23, 42, 43). To confirm that mitophagy is involved in the anti-M1 polarization effect of taurine, lysosomes were evaluated. We found that after LPS/IFN-γ treatment, red fluorescently-labeled lysosomes increased in the field of view. However, this phenomenon was not observed after taurine supplementation (**Figure 4A**). We then demonstrated the occurrence of mitophagy *via* the co-localization of mitochondria (green) with lysosomes (red). M0 macrophages were found to be polarized into M1, and the red fluorescence of lysosomes changed from scattered dots to dense masses, while the green fluorescence of mitochondria mostly changed from agglomerated masses to granular green fluorescence. The orange fluorescence increased significantly, and the degree of co-localization increased. However, taurine supplementation significantly reduced the colocalization of mitochondria and lysosomes (**Figures 4B, C** **)**. Using the M1 group as a reference signal, we found that the co-localization of lysosomes and mitochondria was reduced after supplementation with taurine (**Figure 4D**).

To further confirm the inhibitory effect of taurine on mitophagy during M1 polarization, we measured the expression of LC3 II/LC3 I, the former is a lipidized form of LC3, which promotes the formation of autophagosomes (44). As shown in the results, compared with M0, the LC3 II/I of M1 polarized macrophages increased significantly. However, LC3 II/I decreased after supplementation with taurine (**Figures 4E, F** **)**. In contrast, p62, as a link between LC3 and ubiquitinated substrates, showed a downward trend in M1, while taurine supplementation increased its expression (**Figures 4E, G** **)**. Importantly, BAF (10 nM) was added in the last 12 h of the treatment time to interfere with the degradation of autophagosomes to observe changes in autophagic flux, the autophagy inhibitor-induced increase of LC3 II accumulation and reduction of p62 degradation were relatively significant upon LPS/IFN-γ treatment alone (**Figures S2A–C**). Second, the expression level of Beclin1, a key protein regulating autophagosome formation, was consistent with that of LC3 II/I, however, a clear trend was not observed (**Figures 4H, I** **)**. Combining these data suggests that taurine blocks the increase in mitochondrial autophagic flux induced by LPS/IFN-γ, which may be related to the formation of autophagosomes.

Mitophagy is mainly regulated by PINK1 and Parkin. PINK1 is believed to accumulate on depolarized mitochondrial membranes and promote mitophagy by recruiting Parkin (45, 46). In addition, recent studies have shown that VDAC1 upregulation or oligomerization can promote the opening of mPTP and the loss of MMP, thereby promoting PINK1/Parkin autophagy/mitophagy (47–49). Our results showed that the expression of VDAC1 and PINK1 increased after LPS/IFN-γ treatment, and taurine supplementation decreased their expression in a dose-dependent manner (**Figures 4J–L**). However, the expression of Parkin was not consistent with that of the other two proteins. Compared with the M0 group, and contrary to our expectations, the expression of Parkin in the M1 group was significantly decreased (**Figures 4J, N** **)**. Since the results presented static changes after 48 h, it is unclear whether the expression of Parkin was consistently low after LPS/IFN-γ treatment. The increased expression of NLRP3 in the model group may provide a potential explanation (**Figures 4M, O** **)**, since studies have shown that NLRP3 can directly promote Parkin degradation through Caspase-1 (50, 51). Taken together, all these data indicate that abundant taurine may prevent mitochondria from being engulfed by blocking VDAC1/PINK1-mediated mitophagy and avoiding the metabolism from tilting to glycolysis required for M1.

### PP2Ac Methylation Is a Positive Regulatory Factor of Taurine on Mitophagy

#### Taurine Weakens PP2Ac Methylation by Regulating the Balance of LCMT-1/PME-1

**Figure 5:**
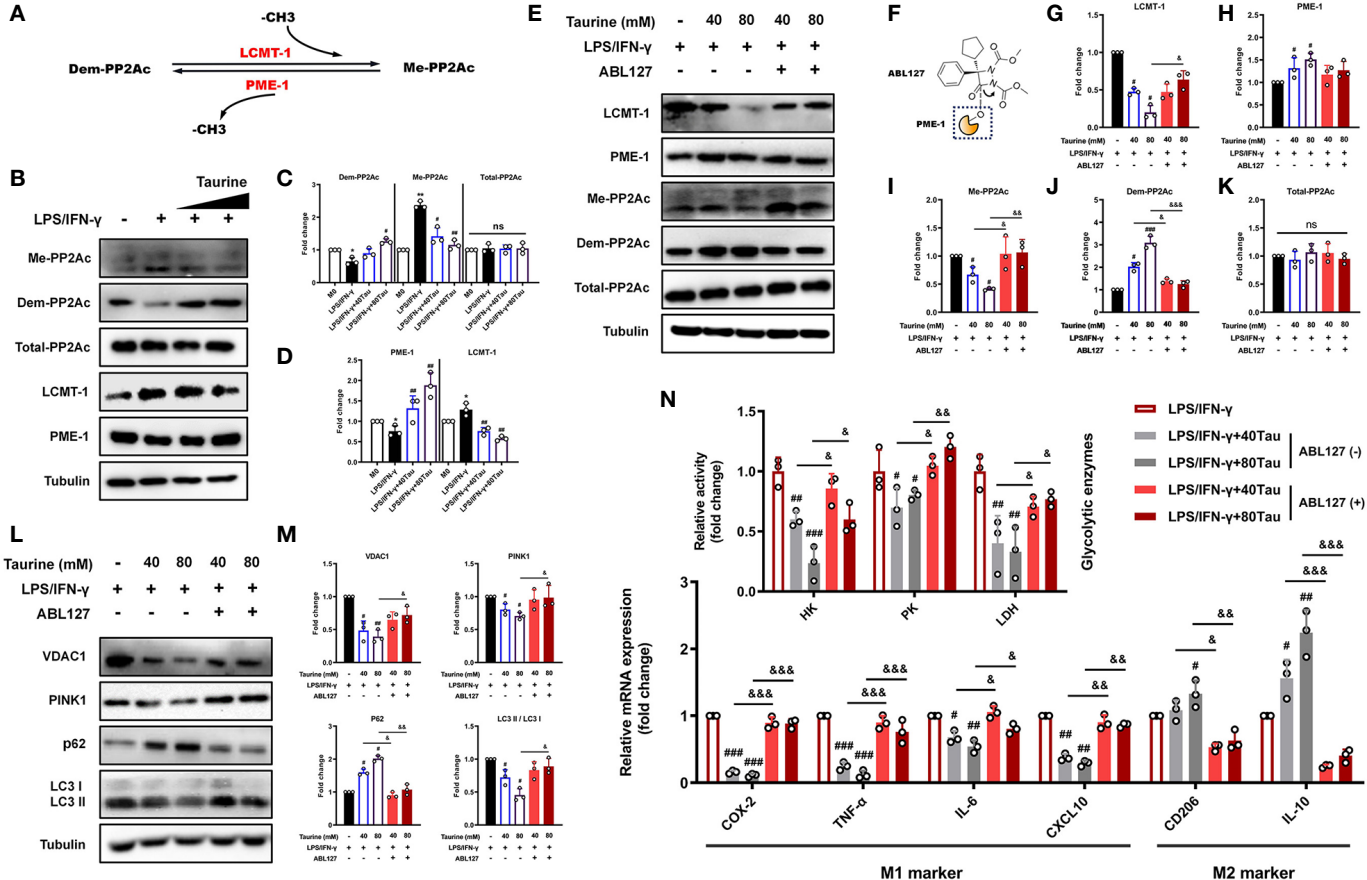
ABL127 prevents the blocking of taurine on mitophagy/glycolysis in LPS/IFN-γ-challenged macrophages. **(A)** Schematic of PP2Ac methylation reaction. **(B)** Taurine inhibits PP2Ac methylation for the representative immunoblots and **(C, D)** quantitation of methylated-PP2Ac, demethylated-PP2Ac, total-PP2Ac, LCMT-1, and PME-1. **(E)** Representative immunoblots and **(G–K)** quantitation of total-PP2Ac, methylated-PP2Ac, demethylated-PP2Ac, LCMT-1, and PME-1 with ABL127 treatment. **(F)** Schematic representation of ABL127 structure. **(L, M)** Representative immunoblots of VDAC1, PINK1, p62, and LC3II/I and its quantitative analysis. **(N)** The glycolytic enzyme activity and the mRNA levels of M1 markers and M2 markers with ABL127 treatment. n=3. *p < 0.05 and **p < 0.01 vs. M0; #p < 0.05, ##p < 0.01 and ###p < 0.001 vs. LPS/IFN-γ; &p < 0.05, &&p < 0.01, &&&p < 0.001 vs. Taurine; ns, p > 0.05.

Recent studies have shown that PP2A activation promotes autophagy (19, 20, 52). The methylation of the catalytic subunit (PP2Ac) regulates the biogenesis of the PP2A holoenzyme, which is closely related to the methyl transfer of LCMT-1 and methyl removal of PME-1 from the bound site (**Figure 5A**) (18, 53). We subsequently evaluated the effects of LPS/IFN-γ and taurine on PP2Ac methylation patterns. The levels of methylated PP2Ac increased and levels of demethylated PP2Ac decreased in LPS/IFN-γ stimulation, accompanied by an increase in LCMT-1 and a decrease in PME-1, suggesting that active PP2Ac methylation signal participated in the pro-inflammatory metabolic adaptation (**Figures 5B–D**). However, taurine supplementation prevented an increase in PP2Ac methylation and upregulated demethylation. We also observed reduced LCMT-1 and elevated PME-1 expression with taurine supplementation, which showed the same trend as PP2Ac methylation and demethylation (**Figures 5B, D** **)**. These data suggest that taurine changes the mode of PP2Ac methylation regulated by LCMT-1 and PME-1 in M1 polarization of macrophages, wherein a lack of PP2Ac methylation may be involved in the failure of M1 polarization caused by taurine supplementation.

#### Maintaining PP2Ac Methylation by ABL127 Blocks the Mitophagy-Mediated Metabolic Reprogramming of Taurine

Our results showed that taurine maintained mitochondrial density by inhibiting mitophagy, adaptively reducing the level of glycolysis and downregulating the M1 polarization level, as PP2Ac methylation. We hypothesized that PP2Ac methylation may function as a positive regulator of the adaptation process (mitophagy) during M1 polarization. To confirm this, ABL127, an inhibitor that activates PP2A by competitively inhibiting PME-1 (**Figure 5F**) (54), was used to specifically upregulate PP2Ac methylation. In line with this, compared with the taurine intervention groups, ABL127 downregulated the level of PP2Ac demethylation and upregulated the level of methylation, accompanied by a change in LCMT-1/PME-1 balance (**Figures 5E–K**). Importantly, ABL127 restored the high expression of VDAC1 and PINK1 in both low and high doses of taurine. The western blotting of LC3 II/I and p62 confirmed that ABL127 increased taurine-blocked autophagic flux (**Figures 5L, M** **)**. This suggests that the regulation of PP2Ac methylation during mitophagy occurs in the early stage of autophagy, that is, the labeling of target organelles. Finally, activity and mRNA assays confirmed that increased PP2Ac methylation upregulated glycolytic enzyme activity, restored M1 marker expression, and downregulated M2 marker expression (**Figure 5N**). Together, these data indicate that a key role of PP2Ac methylation is to promote the metabolic adaptive response of macrophages under external stress, which is accomplished through the positive regulation of mitophagy at an early stage.

### SAM as a Pivotal Determinant of Macrophage M1 Polarization and a New Target of Taurine

**Figure 6:**
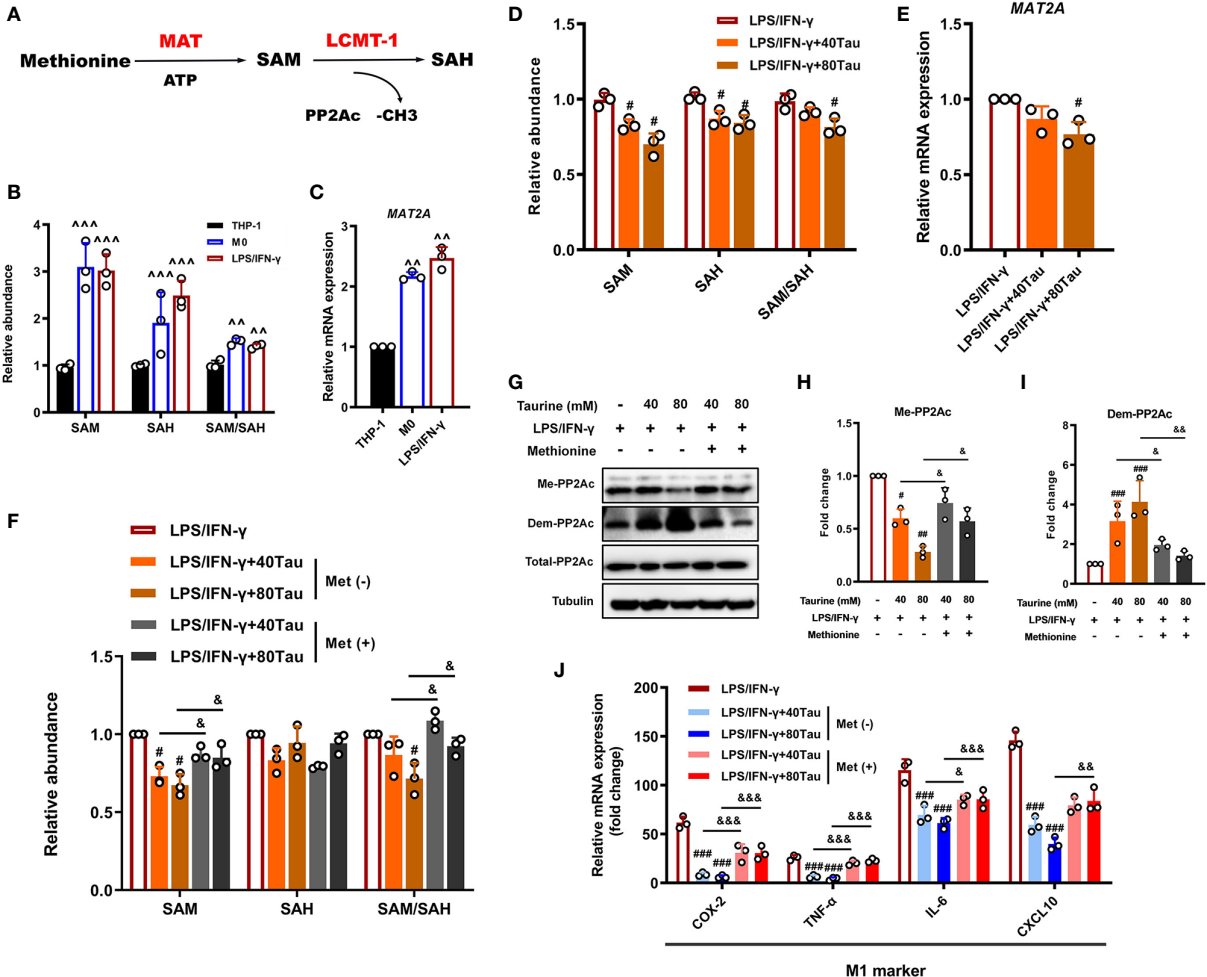
Taurine prevents macrophage M1 polarization by reducing SAM synthesis. **(A)** Schematic representation of the generation of SAM and SAH from methionine and downstream PP2Ac methylation. **(B)** Levels of intracellular SAM and SAH in M1 polarization. **(C)** *MAT2A* mRNA levels in M1 polarization. **(D, E)** Levels of SAM and MAT2a mRNA in taurine-treated macrophages. **(F)** Exogenous methionine restores SAM production inhibited by taurine. **(G)** Representative immunoblots and **(H, I)** quantitation of total-PP2Ac, methylated-PP2Ac, and demethylated-PP2Ac with exogenous methionine. **(J)** mRNA levels of M1 markers with exogenous methionine. n=3. ^^p < 0.01 and ^^^p < 0.001 vs. THP-1; #p < 0.05, ##p < 0.01 and ###p < 0.001 vs. LPS/IFN-γ; &p < 0.05, &&p < 0.01 and &&&p < 0.001 vs. Taurine.

S-adenosylmethionine (SAM) is the most important methyl donor in the body and is synthesized *via* methionine and ATP catalyzed by methionine adenosyltransferase (MAT) (**Figure 6A**). Recent studies have shown that the SAM levels can regulate the methylation patterns of multiple methyl acceptors, such as PP2Ac, in multiple systems (55). The ratio of SAM/SAH is an important metabolic indicator of cell methylation status and can directly affect the activity and expression of methyltransferases (56, 57). To determine whether taurine alters the PP2Ac methylation pattern in M1 polarization by affecting the availability of SAM, we used UPLC-MS to detect the levels of SAM and SAH in the cells. The results showed that, compared with the THP-1 group, the SAM level and the SAM/SAH ratio in M0 cells increased markedly, maintained at a higher level in M1 polarization, and gradually stabilized (**Figure 6B**). However, the addition of taurine reduced the abundance of SAM and the ratio of SAM/SAH in a dose-dependent manner (**Figure 6D**). In addition, mRNA detection showed that taurine significantly downregulated the expression of *MAT2a* compared to the M1 model group (**Figures 6C, E** **)**. These phenomena suggest a potential metabolic feedback inhibition pathway in macrophages. Taurine, one of the final products of transulfuration, inhibits the key step in the methionine cycle synthesis of SAM. Although this is reasonable in theory, to the best of our knowledge, this study is there first to report this finding.

Thus far, we can hypothesize that SAM plays a key role in the establishment of the PP2Ac methylation pattern during macrophage M1 polarization. For further verification, we evaluated the effects of exogenous SAM supplementation through methionine treatment. The results showed that the up-regulation of SAM under methionine treatment effectively offset the inhibition of PP2Ac methylation by taurine and restored the expression of pro-inflammatory genes (**Figures 6F–J**). Thus, our data indicate that SAM may be a direct regulatory target of taurine in the anti-M1 polarization effect.

## Discussion

**Figure 7:**
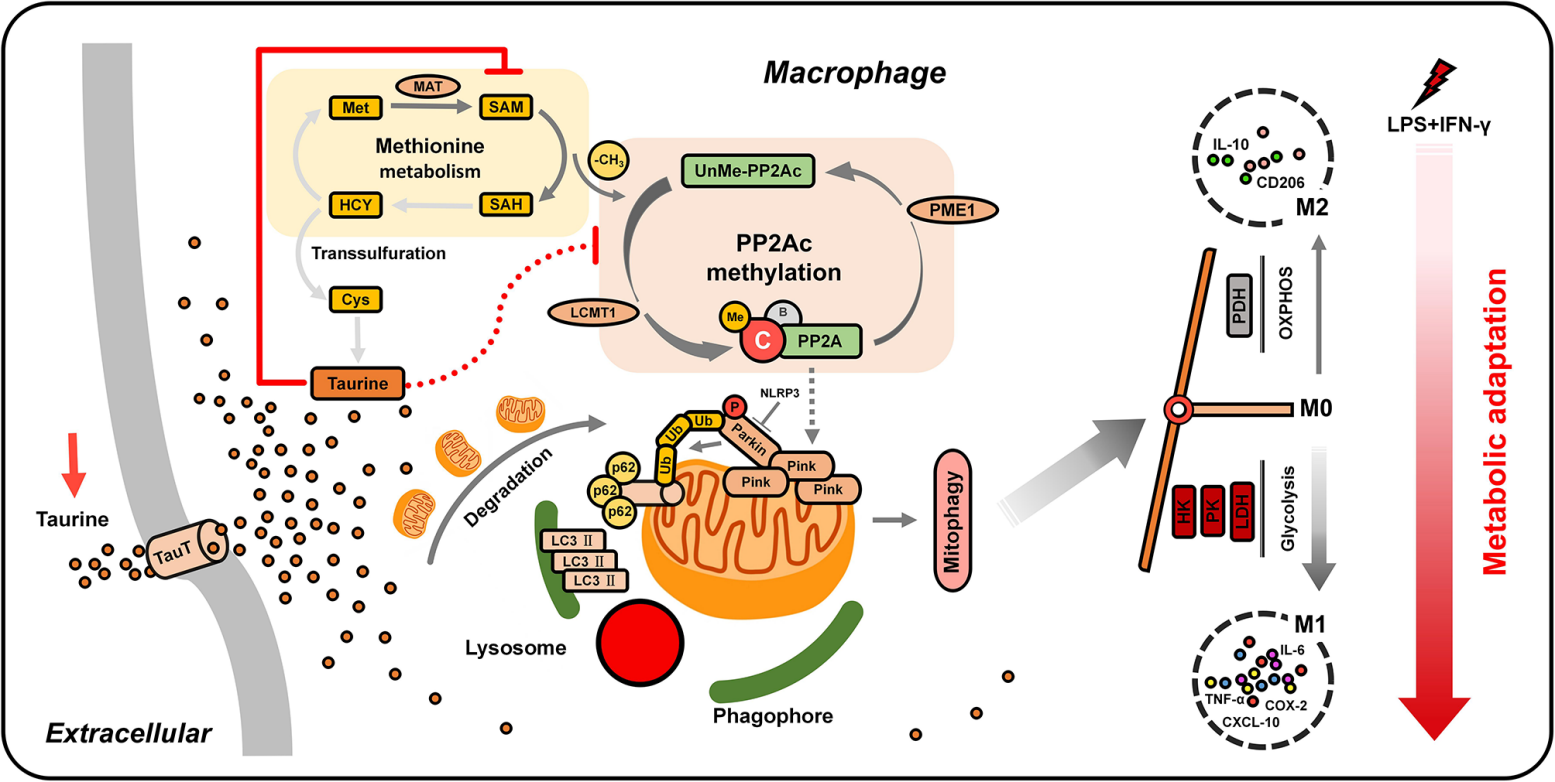
Working model. LPS/IFN-γ-challenged macrophages trigger PINK1-mediated mitophagy by activating SAM-dependent PP2Ac methylation to eliminate mitochondria. This promotes a metabolic switch toward glycolysis, thus realizing the metabolic adaptation of macrophages to pro-inflammatory M1 polarization. The nutrient uptake signal TauT supports the massive influx of taurine after supplementation, which limits the synthesis of SAM. The low availability of SAM is sensed by LCMT-1 and PME-1, hindering PP2Ac methylation. The blocking of PP2Ac methylation negatively regulates the connection between mitochondrial autophagy and glycolysis, ultimately weakening M1 polarization.

The activation of macrophages depends on the adaptation of the metabolism to microenvironmental stimuli. The high plasticity of macrophages determines their biological functions in tissue repair and immune inflammation. Recently, the metabolism has been identified as an important mediator of macrophage function, wherein metabolic changes strongly affect immune function. Studying the regulation mechanism of the metabolic pattern of macrophages is essential for understanding the occurrence and treatment of related-inflammatory diseases (58). In this study, the metabolic adaptability of macrophages was found to play an important role in cell differentiation. We found that the classical activation of macrophages induced by low-dose LPS depended on the clearance of mitochondria by PINK1-mediated mitophagy. Taurine was found to antagonize this process by regulating a series of sophisticated upstream metabolic elements. TauT-mediated taurine uptake was found to block mitophagy by inhibiting SAM-dependent PP2Ac methylation to avoid the metabolic connection to glycolysis, ultimately reducing the excessive pro-inflammatory polarization of macrophages (**Figure 7**). In addition, our data indicate that the methyl donor SAM, an initial regulatory target of taurine, is key for M1 polarization, with its availability playing an active role in ensuring pro-inflammatory metabolic adaptation.

Inflammation is closely related to metabolic diseases, such as obesity and type 2 diabetes (1). The interaction between metabolism and immunity plays a pivotal role in the occurrence and development of such diseases. The actions of macrophages in these pathologies are highly appreciated, as chronic low-grade inflammation caused by the massive infiltration of M1 macrophages usually aggravates the course of disease (59–61). Taurine has been shown to significantly improve inflammation in different physiological and pathological models. For example, the supplementation of taurine in the diet of mice can reduce the expression of inflammatory factors, thereby inhibiting hyperglycemia in an obesity model (35). However, the precise mechanisms by which taurine exerts its anti-inflammatory effects are not yet fully understood. We found that the increased expression of taurine synthase *CDO1*, *CSAD*, and the taurine transporter *SLC6A6* at the transcriptional level under the challenge of LPS/IFN-γ exert resistance against M1 polarization. TauT controls the relative balance of endogenous taurine content according to the needs of different tissues (62). We found that taurine-enriched incubation attenuated LPS/IFN-γ-induced inflammatory cytokine expression, and even shifted macrophages to the M2 anti-inflammatory phenotype. The upregulation of TauT in M1 polarization is a preparation for the next taurine supplement to prevent the continued occurrence of excessive inflammation. Treatment with 40 mM or 80 mM taurine markedly increased the abundance of intracellular taurine However, it is interesting to note that the abundance did not nearly double between the treatment groups, but changed only very slightly. There may be a more precise regulation of taurine homeostasis in macrophages, and the detection of taurine abundance at the final time point is a limitation due to the inability to evaluate the dynamic changes of taurine during treatment.

Energy metabolism plays a vital role in the balance of macrophage polarization and the execution of immune functions (63). The bactericidal ability of activated M1 inflammatory macrophages is derived from the enhancement of glycolysis to provide rapid power (64). In contrast, M2-type polarization related to tissue remodeling and repair requires continuous and large energy supply; therefore, fatty acid oxidation (FAO) and mitochondrial OXPHOS are more suitable for M2-type macrophages (65). We confirmed that glycolytic enzymes HK, PK and LDH in the M1 inflammatory phenotype were hyperactive, and the activity of the rate-limiting enzyme PDH, which regulates the production of acetyl-CoA in the TCA cycle, is down-regulated. These data suggest that the connection to mitochondrial OXPHOS in M1 macrophages is impaired. However, the massive influx of exogenous taurine maintains the active homeostasis between glycolytic enzymes and PDH. The output is a decreased expression of M1 genes and an increased expression of anti-inflammatory genes associated with M2. The tendency of the metabolic pattern determines the direction of macrophage polarization. The growth advantage of M1 conferred by glycolysis was eliminated by taurine. Behind these metabolic events, we found that the most striking feature was large fluctuations in the number of mitochondria, that is, M1 macrophages had an extremely low mitochondrial density, and the taurine group was comparable to the unpolarized ones. In terms of mechanism, this is the direct cause of the transformation of energy metabolism (39). Taurine opens the switch from M1 to M2, which largely depends on the maintenance of mitochondrial number.

Mitochondria are dynamic organelles, and their number and functions can be changed according to the metabolic state required by the cell (66). In the immune system, the homeostasis of mitochondrial function is a prerequisite for host inflammatory response and host defense (67). Mitophagy is a key means of regulating mitochondrial homeostasis. However, the regulation of mitochondrial homeostasis during macrophage polarization remains unclear. A recent study suggested that NIX-mediated mitophagy may be a switch to maintain M1 macrophage-dependent glycolysis. They found that by inhibiting phagocytosis and autophagosome formation, mitochondrial abundance was increased and glycolytic enzyme expression was downregulated, leading to a decrease in the M1 markers TNF, NOS2, and IL1B, accompanied by the appearance of M2-like cell morphology (21, 43). These findings are consistent with our results. We believe that in response to the inflammatory response, mitophagy-induced metabolic kinetic changes are triggered during M1 polarization, which facilitates the connection of metabolism to glycolysis to comply with the energy metabolism required by macrophages for the pro-inflammatory response. This alteration greatly depends on the incompleteness of the TCA cycle after mitochondria are swallowed in large numbers. In addition, we found that VDAC1 and PINK1 may be new members of macrophages that rely on mitophagy to perform metabolic reprogramming, together with NIX. However, we unexpectedly found that the expression of Parkin, which is theoretically closely related to PINK1 changes, was reduced. Despite this, it is worth noting that PINK1 can activate mitophagy alone (68), which successfully drives mitochondria into the autophagy-lysosome program *via* p62-LC3 during M1 polarization. However, considering that the result we detected was the last time point, that is, after 48 h of LPS/IFN-γ treatment, the expression of Parkin may be dynamic. A recent study has shown that the activation of NLRP3 can initiate the hydrolysis of parkin and inhibit mitophagy (50). We also observed a change in NLRP3 expression in our results, which may help to explain the abnormal expression of Parkin. In future research, we will focus on the reasons behind the abnormality of Parkin, which seems to be a balancing mechanism to avoid oversupply of mitophagy.

Little is known about the classical activation diversity of macrophages induced by different doses of LPS. The kinetic mechanism of macrophage phenotypic transition driven by high to low doses of LPS can easily be mistaken as a simplified version. It is worth mentioning that, unlike the activation of autophagy in our model, many studies have shown that LPS blocks the process of autophagy/mitophagy in macrophages, which can be manifested as an upregulation of p62, a decrease in LC3 II, PINK1/Parkin, and lysosomes, or an accumulation of autophagosomes (69–72). However, the LPS doses mentioned in these studies were extremely high compared to ours. Interestingly, in a study of osteoclasts, a type of bone macrophage treated with relatively low doses of LPS (50 ng/mL), was found to activate autophagy (73). Furthermore, we found that mitophagy activation was consistent with the direction of macrophage M1 pro-inflammatory polarization. Other studies have shown that high-dose LPS or other pro-inflammatory mediators can block autophagy, induce the accumulation of defective mitochondria, and decrease oxygen consumption (OCR), resulting in the generation of mitochondrial ROS, which promotes the classical M1 activation of macrophages (74). In contrast, our LPS/IFN-γ stimulation model interprets a special classic activation state driven by mitophagy; only low-density mitochondria are retained in M1 macrophages. This seems to be a top-down, precise programmed mitochondrial clearance that macrophages spontaneously seek metabolic reprogramming in the face of endotoxins. The same way of eliminating redundant mitochondria by mitophagy also exists in the development and differentiation of other cells, such as the transition from beige adipocytes to white adipocytes (22), the maturation of reticulocytes (75), the differentiation of neuroblasts into retinal ganglion cells (43), and the maternal inheritance of mitochondria (76). Therefore, our macrophage pro-inflammatory model is closer to the spontaneous physiological state of cells, and the molecular basis of M1 polarization may be completely different from that of severely injured cells. In this state, the function of a single mitochondria may not be greatly limited, and the changes in the indicators of energy metabolism in our results could be attributed to the sharp decline in the number of mitochondria. Mitophagy eliminates unnecessary mitochondria during differentiation under moderate conditions. Generally speaking, their goal is to continuously adjust their metabolic tendency and approach the most suitable differentiation endpoint. Even though LPS from low to high doses can trigger strong inflammatory reactions in innate immune cells and tissues, the underlying mechanisms are not the same. Our data provide new insights into low-dose LPS-induced macrophage M1 polarization, and more pro-inflammatory mechanisms under different stimulation modes are still worth exploring (74).

In a previous study, PP2Ac methylation was shown to be involved in macrophage-related inflammatory responses, mostly related to the activation of NF-κB and subsequent nuclear transcription. Interestingly, we confirmed here that the reversible PP2Ac methylation state also drives pro-inflammatory activation *via* the mitophagy pathway. In terms of the mechanism, we found that VDAC1 and PINK1 are mediators of PP2Ac methylation involved in mitophagy, and maintaining the hypermethylation of PP2Ac is necessary to induce high levels of expression in both. The VDAC1 levels are related to the frequency of mPTP opening (77), and the opening of the mPTP depolarizes mitochondria to activate PINK1/Parkin-mediated mitophagy (78). In addition, it is worth noting that there is a study suggesting that VDAC1 may promote autophagy at the transcriptional level (49). In short, in the process of M1 polarization, VDAC1 and PINK1 may work synergistically to promote the degradation of mitochondria *via* mitophagy. Our results strongly support the regulatory role of PP2Ac methylation on these two mitochondrial proteins, although it is not clear how it works. We consider that the link between PP2Ac methylation and VDAC1/PINK1 is still largely based on improved substrate dephosphorylation. A recent study has shown that the deletion of serum/glucocorticoid regulated kinase-1 (SGK-1) upregulates VDAC1 abundance *via* dephosphorylation at the Ser104 site and promotes mPTP-dependent mitophagy (49). Furthermore, the inactivation of kinase PDK2 under metabolic stress can activate PINK1-mediated mitophagy by promoting the dephosphorylation of PARL to transform into low-activity PACT (79). PARL acts as a protease that promotes the splicing and degradation of PINK1 in the mitochondrial membrane. There are three phosphorylation sites that regulate PARL activity. In short, dephosphorylation at Ser65, Thr69, and Ser70 promotes β cleavage to convert PARL into PACT (80, 81). This lack of phosphorylation modification supports insufficient degradation of PINK1 and accumulation on the membrane. These findings suggest the potential of PP2A dephosphorylation to promote the initiation and development of mitophagy. In future work, we will focus on the connection between PP2A-related dephosphorylation events and mitophagy. In general, our data suggest that taurine is a key regulator of the hyperpolarization of M1 macrophages. Abundant taurine prevented the accumulation of PINK1 and blocked mitophagy *via* the inhibition of PP2Ac methylation, thereby destroying the metabolic pattern required by pro-inflammatory factors.

SAM plays a key role in maintaining cell homeostasis and cell cycle regulation. Recent studies have shown that SAM drives the activation of pro-inflammatory macrophages by promoting histone H3 lysine 36 trimethylation (13). In this study, we provide a new insight that the levels of SAM, the principal methyl donor in methylation reactions, could be a critical determinant in establishing the PP2Ac methylation patterns of M1 macrophages. We found that during the activation of THP-1 cells into macrophages, the content of intracellular SAM increased markedly, while the expression of SAM synthetase *MAT2A* was only slightly upregulated. After 48 h of LPS/IFN-γ treatment, the expression of *MAT2A* was up-regulated, while the abundance of SAM did not change significantly compared with M0. This is inconsistent with a previous study in which LPS was found to promote the increase of SAM in macrophages (13). However, it is worth noting that the LPS dose used by Yu et al. was 50-times higher than ours. This again shows the difference in the inflammatory response induced by low- and high-dose LPS. We speculate that the activation of methylation consumes SAM continuously, and the upregulation of *MAT2A* promotes SAM homeostasis to fuel the subsequent PP2Ac methylation reaction for M1 polarization. As shown in our results, the intracellular surge of taurine delayed the synthesis of SAM through MAT by methionine and was sensed by PP2Ac to support the remodeling of methylation modification. The high expression of SLC6A6 responsible for taurine uptake may be a self-negative regulation that antagonizes the pro-inflammatory process, wherein the activation of methionine and SAM metabolism and the high availability of SAM create a metabolic advantage for macrophage M1 polarization.

Our study suggests a link between methionine metabolism, PP2Ac methylation, and mitophagy in driving pro-inflammatory phenotypes, and emphasizes the role of energy metabolism in promoting the occurrence of inflammation. We found that taurine acts as a metabolic balancer in the process of M1 polarization induced by low-dose LPS/IFN-γ to prevent excessive pro-inflammatory responses. In general, macrophage M1 pro-inflammatory polarization requires PINK1-mediated programmed mitophagy to trigger and maintain glycolysis, which is mainly positively regulated through sustained PP2Ac methylation driven by sufficient methionine/SAM metabolic flux. This regulates the accumulation of PINK1 in mitochondria, highlighting PP2A as a potential member of the mitophagy pathway. TauT-mediated taurine influx initially dragged SAM synthesis to negatively regulate the above process and reprogram the polarized phenotype of macrophages. Our data reveal the underlying mechanism of taurine-coupled macrophage energy metabolism and suggest that PP2Ac methylation may be a feasible target for the treatment of chronic inflammatory diseases related to macrophage M1 polarization. Further studies on primary human myeloid cells will be needed to confirm a similar metabolic basis and molecular mechanism.

## Data Availability Statement 

The raw data supporting the conclusions of this article will be made available by the authors, without undue reservation.

## Author Contributions

LM performed most of the experiments, analyzed the data, and drafted the manuscript. CLL provided support with the statistical analysis and experimental technique. BW drafted the art work and manuscript and made contributions to interpretation of the results. CHL, LMM, CC, and XW participated in the experiment and data analysis. NZ helped set up SAM/SAH assay. LL, QW, and XZ critically revised the manuscript. ST and XL conceived the study, developed the experimental design, and analyzed and interpreted the data. All authors contributed to the article and approved the submitted version.

## Funding

This work was supported by the Natural Science Foundation of Guangxi Province [grant numbers 2018GXNSFAA281130] and the National Natural Science Foundation of China [grant numbers 81860585, 81460506 and 81760576].

## Conflict of Interest

The authors declare that the research was conducted in the absence of any commercial or financial relationships that could be construed as a potential conflict of interest.

## Supplementary Material

Supplementary Figure 1 The effect of taurine supplementation on M1/M2 markers in M0 macrophages. After M0 macrophages were treated with or without two different concentrations of taurine for 48 h, the mRNA expression levels were detected and quantified. n=3.

Supplementary Figure 2 Induced autophagic flux is blocked by taurine in LPS/IFN-γ-challenged macrophages. **(A–C)** Representative immunoblots and quantitation of LC3 II/I and p62 in macrophages incubated with LPS/IFN-γ and taurine alone or co-incubated in presence or absence of 10 nM BAF (last 12 h). n=3. *p < 0.05 and ***p < 0.001 vs. BAF absence. **(D)** Changes of mitophagy-related protein expression at different time points during M1 macrophage polarization (***Module D belongs to another unpublished paper of this research group***).
