# Supplementary figures and images for: Taurine Antagonizes Macrophages M1 Polarization by Mitophagy-Glycolysis Switch Blockage via Dragging SAM-PP2Ac Transmethylation
Source: Front Immunol. 2021 Apr 12;12:648913. doi: 10.3389/fimmu.2021.648913 (PMC8071881; doi:10.3389/fimmu.2021.648913)

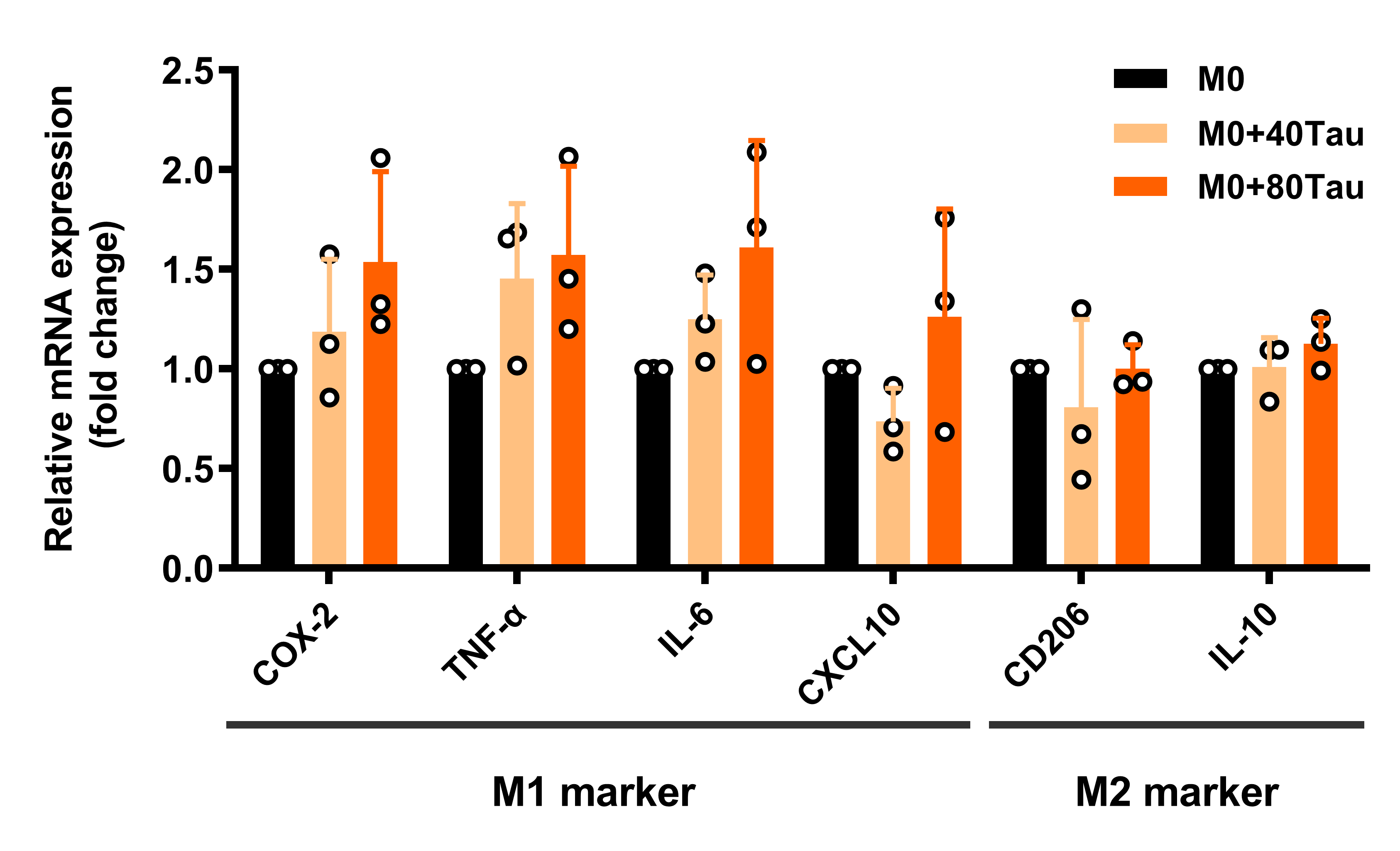

Supplement: Supplementary Figure 1 — The effect of taurine supplementation on M1/M2 markers in M0 macrophages. After M0 macrophages were treated with or without two different concentrations of taurine for 48 h, the mRNA expression levels were detected and quantified. n=3. [file Image_1.png]

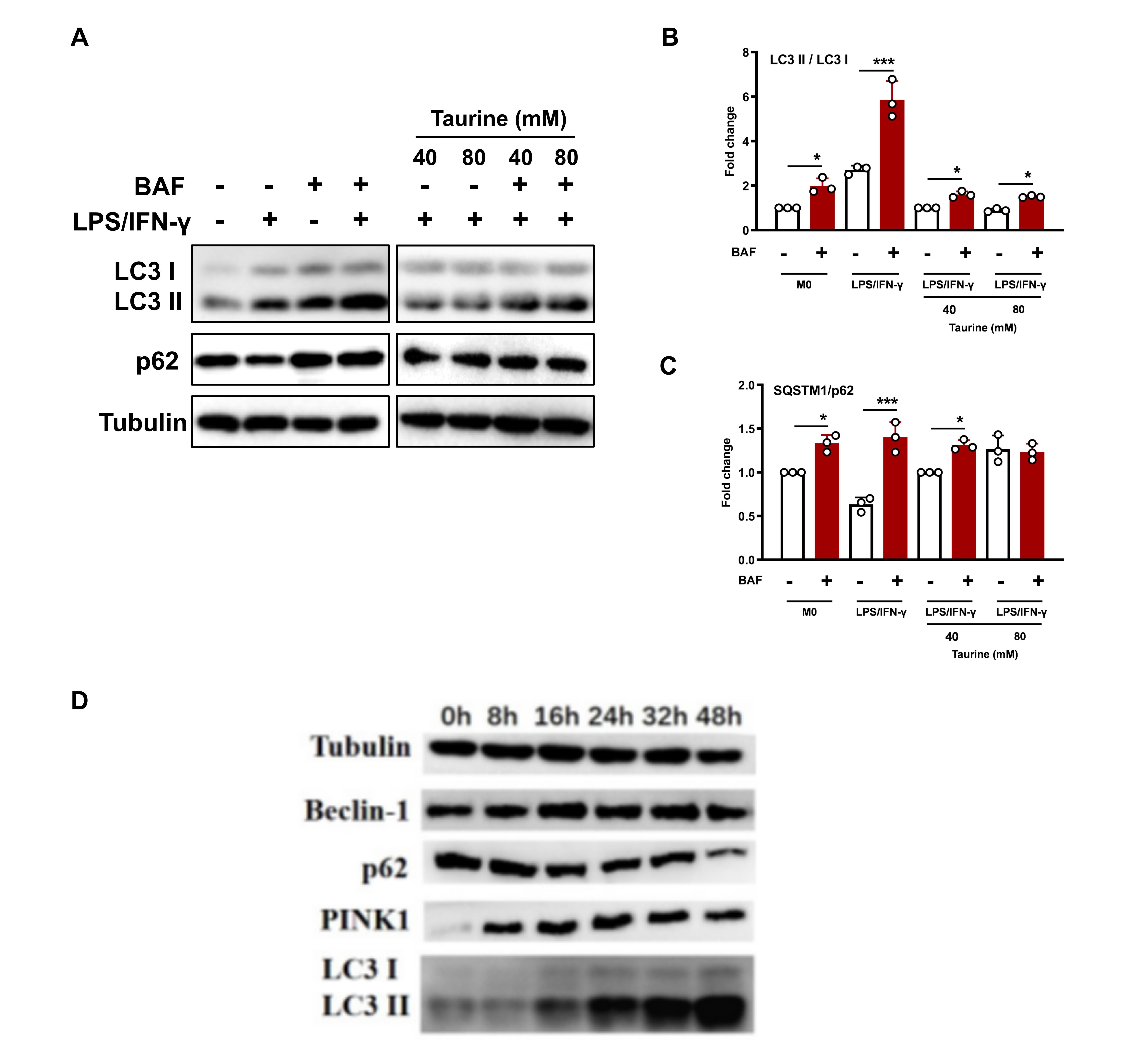

Supplement: Supplementary Figure 2 — Induced autophagic flux is blocked by taurine in LPS/IFN-γ-challenged macrophages. (A–C) Representative immunoblots and quantitation of LC3 II/I and p62 in macrophages incubated with LPS/IFN-γ and taurine alone or co-incubated in presence or absence of 10 nM BAF (last 12 h). n=3. *p < 0.05 and ***p < 0.001 vs. BAF absence. (D) Changes of mitophagy-related protein expression at different time points during M1 macrophage polarization (Module D belongs to another unpublished paper of this research group). [file Image_2.jpeg]
